# Bibliometric analysis of the association between air pollution and allergic rhinitis

**DOI:** 10.1080/16549716.2025.2547434

**Published:** 2025-09-10

**Authors:** Zhigang Geng, Yuqiang Ma, Xueping Qi

**Affiliations:** a Department of Otolaryngology, Head & Neck Surgery, Shanxi Medical University Second Affiliated Hospital, Taiyuan, Shanxi Province, China; b Department of Otolaryngology, Children’s Hospital of Shanxi Province, Taiyuan, China

**Keywords:** Bibliometric, Web of Science, air pollution, allergic rhinitis, visualization

## Abstract

**Background:**

Allergic rhinitis (AR) is an increasingly prominent global public health issue, where air pollution significantly contributes to its rising incidence. Although numerous studies have explored the link between air pollution and AR pathogenesis, comprehensive summaries are still limited.

**Objective:**

This study performs a bibliometric analysis to identify research hotspots and emerging trends, offering insights into AR prevention and management.

**Methods:**

Literature related to on air pollution and AR was retrieved from the Web of Science Core Collection database. Visualization tools, including VOSviewer, CiteSpace, and Bibliometrix R, were utilized to analyze contributions by countries, institutions, authors, journals, and keywords, with the aim of predicting future research trends.

**Results:**

A total of 4,020 authors, 1,368 institutions, and 75 countries contributed to 753 publications. The United States leads in research contributions, while China has shown rapid growth since 2012. Prominent authors include Deng Qihong and Lu Chan have made significant contributions. Keyword analysis revealed five major clusters: Asthma and Allergic Diseases, Environmental Factors, Climate Change and Exposures, Epidemiology and Risk Factors, and Population-Specific Research. Key topics covered include atopy, childhood asthma, climate change, pollution exposure, and air pollutants.

**Conclusion:**

This first bibliometric analysis of air pollution and AR highlights a strong link between air pollution and AR pathogenesis. Enhanced environmental controls and air quality monitoring are essential for AR prevention. However, the complex composition of air pollutants presents challenges in elucidating specific mechanisms.

## Paper Context

**Main findings:** Air pollutant exposure has been identified as a significant risk factor contributing to the progressive rise in allergic rhinitis prevalence. As the first bibliometric analysis of the air pollution-Allergic rhinitis relationship, it shows contributions and theme evolution, enriching the research framework. **Added knowledge:** Our study identified 75 countries, 4,020 authors, and 1,368 institutions’ global participation, with the US and China leading. Research grew notably from 2005-2011 and after 2010, and five keyword clusters clarified research focuses.**Global health impact for policy and action:** It also proves environmental governance and air quality monitoring are crucial for Allergic rhinitis prevention, with European strategies as models for global policy-making to relieve Allergic rhinitis epidemic pressure.

## Background

In recent decades, the acceleration of economic development coupled with progressive environmental deterioration, has elevated allergic disorders to a critical global public health challenge. Epidemiological data reveal that allergic rhinitis (AR) currently affects approximately 30%-40% of the population, with over 500 million confirmed cases worldwide. Notably, about 20% of AR patients present with comorbid asthma, thereby imposing substantial socioeconomic burdens due to a diminished quality of life and increased healthcare expenditures [1,2]. Although AR is recognized as a disease with genetic predisposition [3]; the human genome has maintained remarkable stability over the past half-century. This evolutionary persistence contradicts the observed epidemiological rise in AR incidence, strongly suggesting the involvement of critical environmental cofactors. The evidence implicates air pollution as a major environmental threat contributing to the pathogenesis of various human diseases, particularly respiratory conditions [4]. The World Health Organization(WHO) estimates that air pollution is responsible for approximately 3 million annual deaths globally, which represent 5% of total mortality and demonstrate significant associations with cardiovascular pathologies, respiratory disorders, and immune-mediated allergic diseases [5]. These findings underscore the need to elucidate how airborne pollutants drive AR pathogenesis, such knowledge critical for developing innovative prevention and treatment strategies for this global health burden.

Growing evidence suggests that air pollution, such as tobacco smoke, ozone, nitrogen dioxide, inhalable particulate matter(PM), allergens, and occupational exposure, contributes to the pathogenesis of AR [6,7]. A survey on childhood allergies has demonstrated a significant correlation between the frequency of rhinitis exacerbations and air pollutant concentrations. Additionally, this study further revealed that prolonged exposure duration is independently associated with elevated risks of pediatric respiratory atopic disorders [8], suggesting cumulative dose-response relationships in allergic pathogenesis. A longitudinal cohort study enrolling children of parents with documented sensitivity to airborne allergens demonstrated a significant association between prenatal and early-life exposure to diesel exhaust particles (DEP) and elevated odds of early-life sensitization. The prevalence of sensitization was positively correlated with allergen-specific wheal diameters measured at ages two and three, corroborating dose-dependent immune responses during critical developmental periods [9]. Notably, a positive correlation exists between air pollution and increased incidence of AR and related symptoms in high-traffic urban areas. Exposure to pollutants directly affects the immune system by inducing inflammatory responses through oxidative stress and altering protein immunogenicity [10]. Taken together, air pollution is a significant risk factor for the onset and exacerbation of AR; therefore, a comprehensive clarification of the association between air pollution and AR is crucial for formulating targeted prevention and control strategies.

Bibliometrics, as an objective quantitative analysis tool, has been widely applied across various fields and its core advantage lies in revealing the evolutionary patterns, hotspot clusters, and potential gaps in research fields through objective data (such as the number of publications, keyword co-occurrence, and collaboration networks), thereby avoiding biases in subjective analysis [11]. For example, existing studies have systematically analyzed global research trends regarding air pollution and asthma using bibliometrics, with key research areas focusing on air quality, exposure PM2.5, and asthma exacerbation [12]. Other studies have conducted bibliometric analyses on air pollution and chronic obstructive pulmonary disease (COPD), clearly demonstrating the leading position of European and American countries in this field as well as the research frontier of ‘atmospheric fine PM2.5 and airway remodeling’ [13]. Notably, there have been no bibliometric analysis on ‘air pollution and AR’ to date. Therefore, this study is the first to focus on the field of ‘air pollution and AR’ that has not been systematically analyzed. Its value lies not only in integrating existing research findings but also in revealing research patterns and gaps from a quantitative perspective, thereby providing a scientific basis for collaborative innovation and translational application in this field.

## Methods

### Citation data collection

We retrieved relevant articles on air pollution and AR from the Web of Science database, including all papers published in this field before 1 July 2024. The search strategy combined the following terms: TS=(‘Air pollution’ OR ‘air pollutants’) AND TS=(‘allergic rhinitis’ OR ‘rhinitis, allergic’). The inclusion criteria specified English-language documents restricted to ‘Article’, ‘Review’, or ‘Book Chapter’ types. All data utilized were directly downloaded and analyzed without additional processing, eliminating the need for ethical approval as no human or animal subjects were involved.

### Bibliometric and visualization analysis

Eligible studies were downloaded from the Web of Science(WOS) Core Collection and incorporated with citation reports to analyze publication outputs, encompassing country/region distributions, author collaborations, journal contributions, institutional productivity, highly cited papers, and keyword co-occurrence patterns (Self-citations were not removed from these entries). Bibliometric software packages (VOSviewer, CiteSpace, and Bibliometrix R) were employed to conduct systematic and integrative analysis of the literature, generating visual representations of bibliometric networks. This approach facilitated the identification of research frontiers in air pollution-AR relationships and provided evidence-based insights for prioritizing future research directions.

## Results

### Publication trends of global literature

**Figure 1.**
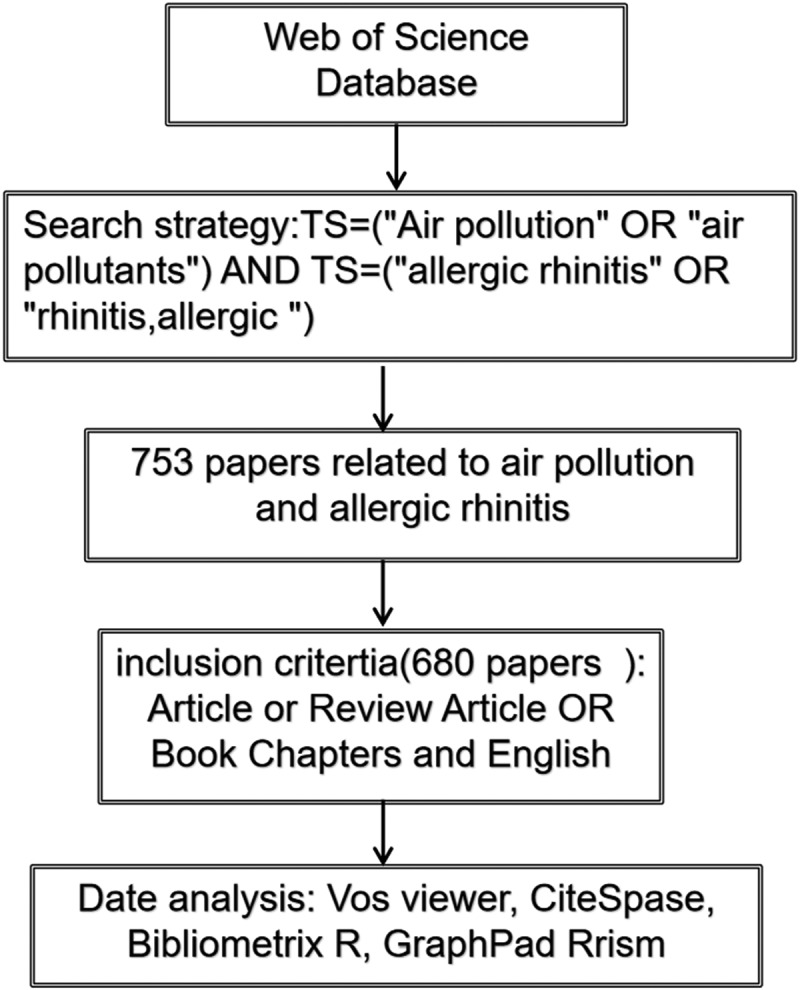
Screening procedures related to air pollution and allergic rhinitis based on Web of Science.

**Figure 2.**
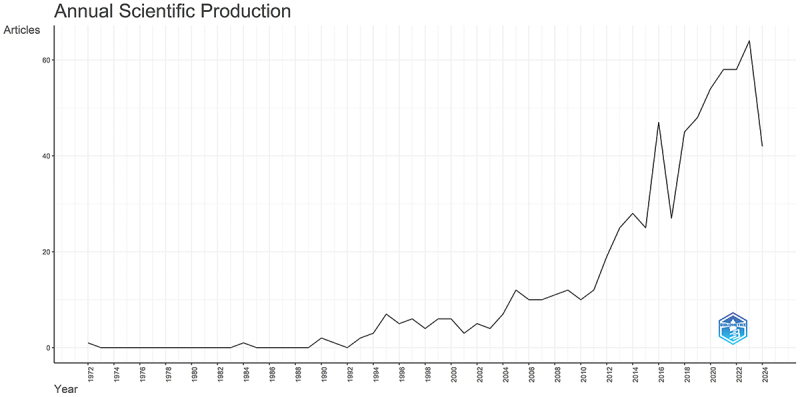
Number of global publications related to air pollution and allergic rhinitis.

Our systematic search strategy resulted in 753 relevant publications (566 articles and 148 reviews) in the Web of Science database ranging from 1972 to July 2024. Following the exclusion of non-English documents, 680 records were retained for final analysis (Figure 1). Research on air pollution and AR was limited before 2004, increased significantly from 2005 to 2011, and has shown a rapid year-on-year growth trend since 2010, with 64 publications in 2022 (Figure 2). These bibliometric trends reflect rising scholarly interest in air pollution-AR relationships, suggesting a trajectory of continued emphasis in future research.

### Analysis of publication volume by country/region

A total of 75 countries/regions have contributed to this research domain. The United States topped the list with 174 publications (H-index = 44), followed by China (159 publications, H-index = 38) and South Korea (60 publications, H-index = 23). The H-index, a metric reflecting both publication quantity and quality, was employed to evaluate scientific impact [14].

**Figure 3.**
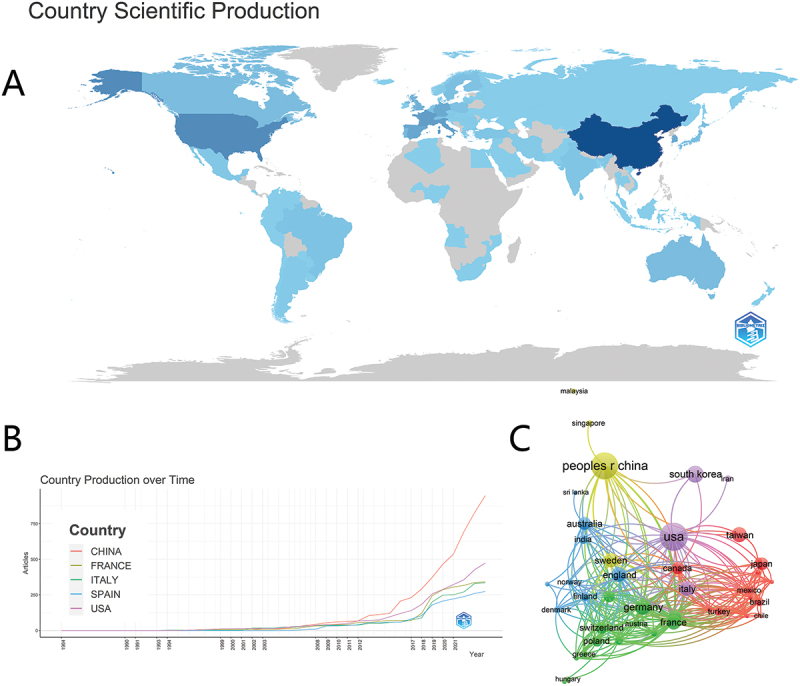
Number of publications and cooperation on air pollution and allergic rhinitis by country. (A) Map of the distribution of publications by countries/regions; (B) the growth trend of the number of publications in the top five countries; (C) the coupling of the number of national publications by VOSviewer.

Geospatial mapping generated via R Studio revealed the regional clustering of research activity in China, the United States, and France based on publication density (Figure 3A). A remarkable finding was the exponential growth of Chinese contributions to this field after 2012 (Figure 3B). The analysis using VOS Viewer software analysis identified Germany, the United States, and the United Kingdom as the top three countries in research collaboration (Figure 3C). Despite Germany having a lower publication output (*n* = 48), its coupling strength was the highest at 172, exceeding the United States (171) and the United Kingdom (146). In the visualization, nodes represented the publication volume of each country, while lines indicated the associative strength between the research outputs of different countries [15].

### The most productive authors and institutions in the field

**Figure 4.**
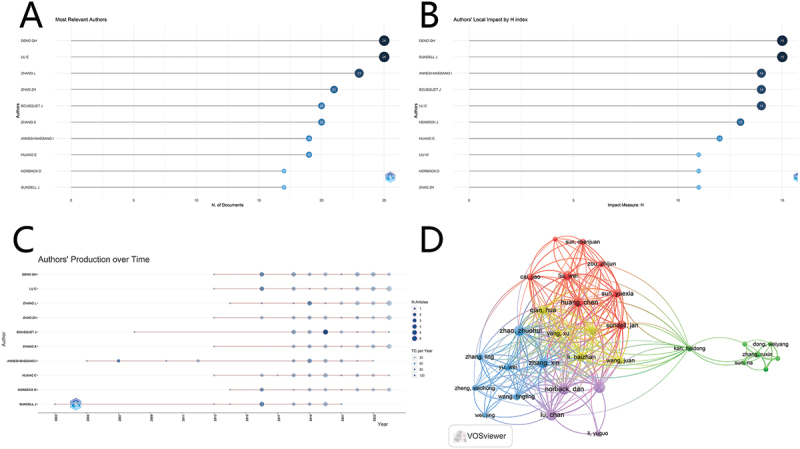
The top 10 authors on research related to air pollution and allergic rhinitis. (A) Number of publications; (B) the author’s H-index; (C) Authors’ production over time. (D) Coupling between authors by VOSviewer.

Currently, 4,020 authors have published research findings in this field. Two prominent authors, Deng QH and Lu Chan, each have 25 publications, with H-indexes of 15 and 14, respectively. Both initiated their publication endeavors in this field in 2013 and have steadily augmented their output since then. Sundell J, who published the first paper in this field in 2003, has a total of 17 publications and a relatively high H-index of 15. Figure 4 illustrates the publication output (A), H-index (B), and Authors’ Production over Time (C) for the top 10 authors. It should be noted that, despite potential minor variations in analysis results from different software, Norback Dan from the Disciplinary Domain of Medicine and Pharmacy at Uppsala University has also made remarkable contributions in this field.

The VOS viewer coupling results indicate that the coupling strengths are 206 for Zhao Zhuohui, 202 for Zhang Xin, 200 for Norback Dan, 190 for Huang Chen, and 185 for Deng Qihong, reflecting the degree of association among these authors these authors (Figure 4D).

**Figure 5.**
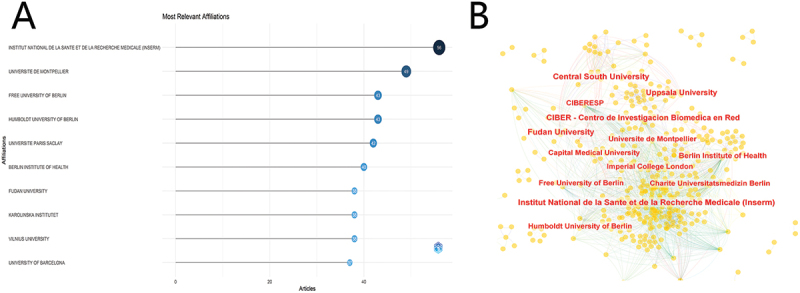
Institutions for research on air pollution and allergic rhinitis. (A) Number of publications by the top 10 organizations. (B) the partnerships between organizations.

A total of 1,368 institutions are engaged in research within this field. The publication output of an institution can be regarded as an indicator of its research competitiveness. As depicted in Figure 5, the Institut National de la Santé et de la Recherche Médicale (INSERM) leads in publication output of 56 papers (A) and demonstrates the closest collaboration with other institutions (B). Among the top 10 institutions, three are from Germany and two are from France.

### Journal analysis and highly cited papers

**Figure 6.**
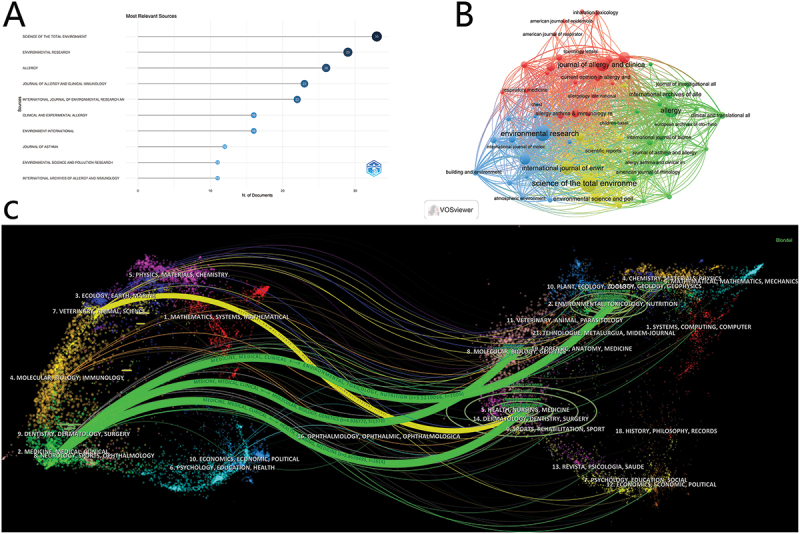
Journals for research on air pollution and allergic rhinitis. (A) Number of publications by the top 10 journals. (B) Coupling between journals by VOSviewer. (C) the dual-map overlay between citing journals and cited journals.

A total of 226 journals have published research on the relationship between air pollution and the incidence of AR. The top 10 journals are presented in Figure 6A. ‘Science of the Total Environment’ has the highest publication output, with 33 papers. When it comes to journal coupling, ‘Environmental Research’ has 29 papers but the highest coupling strength of 6,956. It is followed by ‘Science of the Total Environment’(coupling strength of 6,628) and ‘Environment International’ (coupling strength of 5,353) (Figure 6B).

This study has identified five highly-cited papers in this field, all of which can be accessed online via the WoS website (Table 1). Journal overlay visualization reveals the interdisciplinary relationships within this field. On the left side are the citing journals, while on the right side are the cited journals. The Z-score function quantifies the strength of the relationship between the two, and the colored curves represent the citation trajectory of the reference papers. Figure 6C indicates that the papers are predominantly published in the fields of ecology, materials, immunology, and environmental sciences.

**Table 1.** The list of highly-cited papers on air pollution associated with AR.

Rank	Highly cited documents	Author	Source	JCR	IF	year	Citations
1	Air Pollution and Noncommunicable Diseases A Review by the Forum of International Respiratory Societies’ Environmental Committee, Part 2: Air Pollution and Organ Systems	Schraufnagel DE	Chest	Q1	9.5	2019	491
2	Allergic rhinitis	Bousquet J	NATURE REVIEWS DISEASE PRIMERS	Q1	76.9	2020	360
3	Environmental Exposures and Depression:Biological Mechanisms and Epidemiological Evidence	Van den Bosch M	ANNUAL REVIEW OF PUBLIC HEALTH	Q1	21.4	2019	131
4	Epithelial barrier hypothesis:Effect of external exposome on microbiome and epithelial barriers in allergic disease	Sozener ZC	Allergy	Q1	12.6	2022	152
5	The Evolving Epidemiology of Nasopharyngeal Carcinoma	Chang ET	CANCER EPIDEMIOLOGY BIOMARKERS PREVENTION	Q1	3.7	2021	158

**Figure 7.**
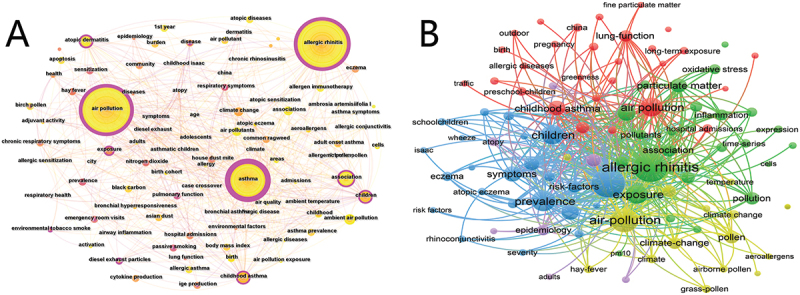
Keyword analysis for research on air pollution and allergic rhinitis. The size of nodes maps the frequency of keyword occurrences, and the lines connecting them represent keyword co-occurrence relationships: the larger the node, the more attention the keyword receives; the thicker the line, the closer the connection between keywords. (A) Co-occurrence analysis of all keywords. (B) Key words cluster analysis, different colors represent different research themes.

Keyword analysis is of particular importance for rapidly discerning research hotspots witin a field and tracing the disciplinary development trends. The top 5 keywords in this field are ‘Allergic rhinitis’, ‘air pollution’, ‘asthma’, ‘children’, and ‘association’(Figure 7A). Be employing VOSviewer for co-occurrence analysis, five principal clusters were identified: Asthma and Allergic Diseases (red), Environmental Factors (green), Climate Change and Exposures (yellow), Epidemiology and Risk Factors (blue), and Population-Specific Research (purple) (Figure 7B). Asthma and Allergic Diseases cluster around clinical perspectives, with common keywords encompassing childhood asthma, eczema, AR, and allergic diseases. Environmental Factors cluster focuses on pollutant source tracing and characteristics, with common keywords including PM10, fine particulate matter, and traffic, reflecting ongoing attention to specific pollutants and their sources. Climate Change and Exposures cluster focuses on macro-environmental associations, with common keywords including climate change, long-term exposure, and temperature. This indicates a growing concern about broader environmental drivers (such as climate change) and their potential impacts on allergic diseases. Epidemiology and Risk Factors cluster centers on disease pathogenesis, with common keywords including pollutants, inflammation, and oxidative stress, focusing on exploring the risk mechanisms underlying disease occurrence and progression. The Population-Specific Research cluster targets vulnerable groups, with common keywords consisting of preschool-children, pregnancy, and birth, focusing on conducting precision research on special populations.

**Figure 8.**
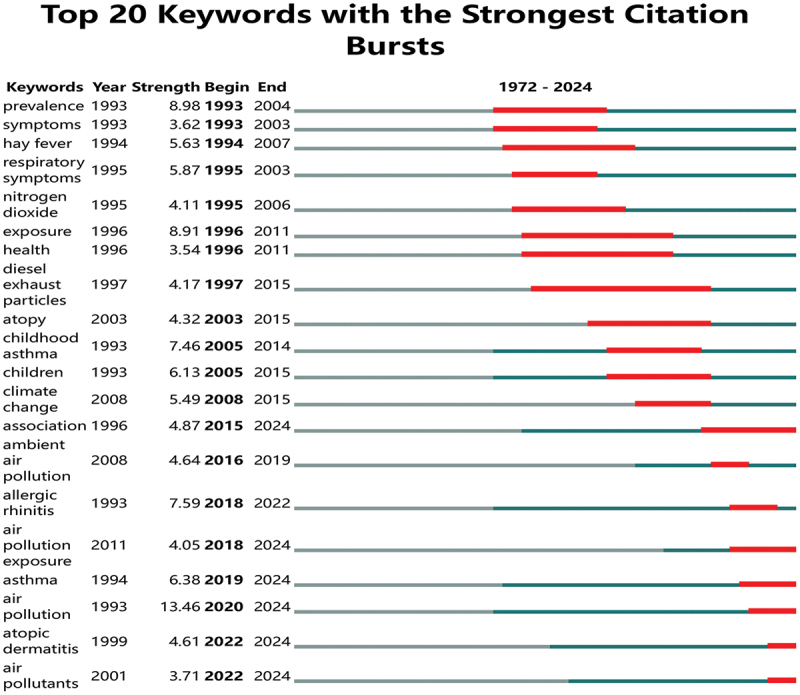
Top 20 keywords with the strongest citation bursts of publications.

Keyword mutations can unveil shifts in research hotspots and emerging trends. The blue lines denote the time interval, while the red lines signify the duration of the hotspot. Figure 8 presents the 20 most frequently-cited keywords. In terms of research trend evolution, this field exhibits clear phased characteristics. During the descriptive stage (1990s), studies focused on disease burden monitoring, centering on ‘symptoms’ and ‘prevalence,’ and initially outlined the apparent associations between PM2.5 and diseases such as asthma. Entering the exposure analysis stage (early 21st century), research delved into pollution source analysis (e.g. ‘NO₂ (nitrogen dioxide)’) and exposure pathways, attempting to unravel the linkage chain between environmental exposure and health impacts. Moving to the integration stage (2010s to present), more systematic breakthroughs have been achieved: it has incorporated ‘climate change (climate change drivers)’ and conducted research on multi-pollutant synergy, such as ‘ambient air pollution.’ This aligns fully with the shift from fragmented research to an integrated paradigm of ‘environmental exposure – biological mechanisms – population health – climate associations.’ The current burst terms ‘ambient air pollution’ and ‘air pollution exposure’ (2011-present) further echo this integration trend, underscoring the research’s role in supporting practical applications. They call for formulating climate-health coordinated policies (e.g. linking pollution and pollen season warnings) and advancing cross-domain collaboration in environmental and public health governance.

## Discussion

As urbanization accelerates, air pollution has emerged as a pressing global social issue that demands immediate attention, and its adverse impacts on human health are attracting growing concern. Air pollution has become one of the leading causes of premature death worldwide. The airway mucosa, a crucial physical barrier in the human body, is significantly affected by air pollution (including microorganisms, allergens, pollutants, etc.) in terms of maintaining internal environmental stability [16,17]. The global incidence of respiratory diseases such as AR and asthma is growing rapidly, with air pollution regarded as a major driving force behind this trend. To comprehensively and objectively assess the current state and future trends of research on the relationship between air pollution and AR, we employed bibliometric analysis to examine papers published prior to 20 July 2024. The results indicate that research in this field has exhibited a clear upward trend, suggesting that greater research efforts will be directed towards this area in the future. A total of 680 papers meeting the search criteria were contributed by 4,020 authors from 75 countries globally. The countries with the highest publication outputs are the United States, China, and France. Over the past decade, China has witnessed the most rapid growth in publication volume, while Germany demonstrates the closest collaboration with other countries.

The INSERM stands as the institution with the highest publication output and has rendered remarkable contributions in this field. Research from INSERM has reported that prevalent air pollutants, including PM2.5, ozone, and nitrogen dioxide, are capable of triggering or exacerbating the symptoms of AR [18]. A large-scale epidemiological study has demonstrated that the prevalence of AR can be approximately 20% higher among populations inhabiting regions with subpar air quality. Additionally, the institution has conducted in-depth investigations into the impact of air pollution on the treatment of AR. Their research has confirmed that air pollution can compromise the immune function of AR patients and impair the barrier -protective function of the nasal mucosa. As a result, it heightens the risk of allergies. The symptoms of AR patients are significantly exacerbated, which leads to a substantial increase in the demand for medications. However, due to the intensification of discomfort, patients may reduce their medication intake [19].

China authors Deng Qihong and Lu Chan lead in publication output. Deng QH and Lu Chan have co-authored multiple papers in this field. Deng QH is from the School of Public Health, Zhengzhou University, Zhengzhou, Henan Province, China. Lu Chan (CL) is from the XiangYa School of Public Health, Central South University, Changsha, Hunan Province, China. Their research has identified significant associations between exposure to air pollution during pregnancy and post-birth, household environmental factors (such as renovations and mold), and climatic factors (such as high temperatures and greenery) and health problems such as AR, colds, and asthma in Chinese children and young adults [20–22].

The highly-cited papers in this research domain disclose that air pollution exerts severe impacts on multiple organ systems, notably the respiratory system, and has attracted extensive attention and investigation [23]. Additionally, environmental factors including climate change, pollution, and biodiversity loss hasten the advancement of allergic diseases [24]. These research findings offer crucial new perspectives for health-risk assessment and put forward policy recommendations to tackle these health threats. As a result, they have a profound influence on public-health policies and related research domains [25].

The results of the keyword-clustering analysis reveal that research directions in this field can be classified into five major themes. Although these themes are distinct, they are also interconnected, highlighting the intricate regulatory network and the central role of air pollution in the development of AR. The evolutionary trends of keywords (such as the increasing frequency of ‘atopy’ and ‘climate change’ and the decreasing frequency of ‘hay fever’) to reveal their connections with future research and public health policies. Specifically, the surge in the use of the keyword ‘atopy’ reflects the deepening exploration in academia into the genetic-environmental interactions of atopic diathesis, which is closely related to the global trend of allergic diseases becoming more prevalent among younger populations; the rising frequency of ‘climate change’ echoes the policy attention paid to the pollutant-pollen synergistic effect in international climate reports. Research has shown that traffic-induced air pollution poses a substantial threat to children’s health [26]. Populations residing near busy streets exhibit an elevated prevalence and increased severity of AR and asthma [27,28]. Common air pollutants linked to traffic, such as PM, ground-level ozone, sulfur compounds, and nitrogen compounds, predominantly exert their effects via oxidative stress. When reactive oxygen species (ROS) disturb the antioxidant equilibrium, they damage cell membranes, proteins, and DNA, thereby triggering airway inflammation and hyperreactivity [29]. Ozone induces hyperplasia of airway epithelial cells, facilitating allergen-induced mucous cell metaplasia and initiating inflammatory responses [6,30]. A correlation exists between air pollution and climate change; elevated air pollutants result in higher pollen density and stimulate pollen-releasing cellular molecules, eliciting inflammation in the upper and lower airways [31]. Nitrogen dioxide shows a positive correlation with pollen concentration, while carbon monoxide has a significantly positive correlation with allergen levels [32]. Moreover, reduced wind speed can impede the dispersion of air pollutants, intensifying respiratory irritation symptoms [18,33].

By interpreting the diachronic evolution of clustered themes (e.g. the prominence of ‘pediatric health’ in Cluster #5), we can establish connections with practical policy needs: on the one hand, ‘preschool-children’ (the purple cluster in Figure 7B) has become a research hotspot, which directly responds to the initiative concerning early exposure intervention in the WHO Guidelines for Children’s Environmental Health; on the other hand, the rise of ‘environmental exposure’ (the yellow cluster) echoes policies such as the EU’s ‘Zero Pollution Action Plan’. Research has confirmed that effective policy adjustments can reduce air pollution, thereby having a positive impact on the prevention and treatment of AR [34]. European governments promote the use of economically and environmentally friendly renewable clean energy to replace fossil fuels and gradually phase out coal-power generation, substantially reducing air pollutant emissions and contributing to air quality control [35]. Raising driving costs and advocating green transportation methods, such as public transit, cycling, and electric vehicles, can help reduce exhaust emissions [36]. Regular indoor ventilation and air filtration, which help prevent mold growth and cockroach breeding, can decrease indoor NO2 concentrations [37]. Furthermore, it is recommended that people plan their outdoor activities wisely, increase their exercise while limiting their exposure during pollen seasons to reduce the incidence and severity of respiratory diseases [38].

### Advantages and limitations

This study utilizes bibliometric and visual analysis methods to examine the literature on air pollution and the incidence of AR, identifying research hotspots and future trends to facilitate further investigation and application of findings. However, this research has some limitations. First, the literature used is restricted to the WOS database and does not encompass other databases, which may result in publication bias. Additionally, high-quality articles published recently that were not incorporated into the study or those with lower citation frequencies might also introduce analytical bias. Despite these limitations, we endeavored to use various bibliometric software to conduct objective analyses, thereby enhancing the scientific quality and reliability of the study.

## Conclusion

It is evident that air pollution represents a significant threat to human health and is closely associated with the incidence of AR. This study comprehensively and scientifically analyzes, for the first time, the existing research on the relationship between air pollution and AR using bibliometric methods. It offers an in-depth analysis and summary of the national, institutional, author, journal, and hot topic aspects in this field, clarifying that air pollution triggers and exacerbates AR, contributing to the rising incidence of the disease. In the future, strengthening environmental controls and focusing on air quality monitoring will be crucial for the prevention of AR. However, the complexity of air pollutant components poses a significant challenge in further exploring the specific mechanisms by which air pollution contributes to AR. We anticipate collaboration among outstanding scholars in this field from various countries to achieve new breakthroughs and provide new insights and strategies for the prevention and treatment of AR.

## References

[1] Hänninen R, Murtomäki A, Svärd F, et al. Being born in autumn or winter is associated with asthma and allergic rhinitis in Finland. Clin Transl Allergy. 2024;14:e12383. doi: 10.1002/clt2.12383 39031968 PMC11259556

[2] Bousquet J, Hellings PW, Agache I, Amat F, Annesi-Maesano I, Ansotegui IJ, et al. Allergic rhinitis and its impact on asthma (ARIA) phase 4 (2018): change management in allergic rhinitis and asthma multimorbidity using mobile technology. J Allergy Clin Immunol. 2019;143:864–14. doi: 10.1016/j.jaci.2018.08.049 30273709

[3] Kay AB, Mackay IR, Rosen FS. Allergy and allergic diseases. First of two parts. N Engl J Med. 2001;344:30–37. doi: 10.1056/NEJM200101043440106 11136958

[4] Vimercati L, Gatti MF, Baldassarre A, Nettis E, Favia N, Palma M, et al. Occupational exposure to urban air pollution and allergic diseases. Int J Environ Res Public Health. 2015;12:12977–12987. doi: 10.3390/ijerph121012977 26501303 PMC4627011

[5] Holnicki P, Tainio M, Kałuszko A, et al. Burden of mortality and disease attributable to multiple air pollutants in Warsaw, Poland. Int J Environ Res Public Health. 2017;14:1359. doi: 10.3390/ijerph14111359 29117145 PMC5707998

[6] Higgins TS, Reh DD. Environmental pollutants and allergic rhinitis. Current opinion in otolaryngology & head and neck surgery. Curr Opin Otolaryngol Head Neck Surg. 2012;20:209–214. doi: 10.1097/MOO.0b013e3283534821 22487789

[7] Naclerio R, Ansotegui IJ, Bousquet J, et al. International expert consensus on the management of allergic rhinitis (AR) aggravated by air pollutants: impact of air pollution on patients with AR: current knowledge and future strategies. World Allergy Organ J. 2020;13:100106. doi: 10.1016/j.waojou.2020.100106 32256939 PMC7132263

[8] Nicolussi FH, Santos AP, André SC, et al. Air pollution and respiratory allergic diseases in schoolchildren. Rev Saude Publica. 2014;48:326–330. doi: 10.1590/S0034-8910.2014048004940 24897055 PMC4206145

[9] Codispoti CD, LeMasters GK, Levin L, et al. Traffic pollution is associated with early childhood aeroallergen sensitization. Ann Allergy Asthma Immunol. 2015;114:126–133. doi: 10.1016/j.anai.2014.10.020 25499550 PMC4308502

[10] Ackaert C, Kofler S, Horejs-Hoeck J, et al. The impact of nitration on the structure and immunogenicity of the major birch pollen allergen bet v 1.0101. PLOS ONE. 2014;9:e104520. doi: 10.1371/journal.pone.0104520 25126882 PMC4134196

[11] Ismail II, Saqr M. A quantitative synthesis of eight decades of global multiple sclerosis research using bibliometrics. Front Neurol. 2022;13:845539. doi: 10.3389/fneur.2022.845539 35280299 PMC8907526

[12] Shu X, Cao J, Liu Q, et al. Global trends and hotspots in the research of the effects of PM2.5 on asthma: a bibliometric and visualized analysis. J Epidemiol Glob Health. 2024;14:1720–1736. doi: 10.1007/s44197-024-00331-4 39625686 PMC11652553

[13] Bouza E, Alvar A, Almagro P, et al. Chronic obstructive pulmonary disease (COPD) in Spain and the different aspects of its social impact: a multidisciplinary opinion document. Rev Esp Quimioter. 2020;33:49–67. doi: 10.37201/req/2064.2019 31933347 PMC6987629

[14] Xiong W, Wang S, Wei Z, et al. Knowledge domain and hotspots predict concerning electroactive biomaterials applied in tissue engineering: a bibliometric and visualized analysis from 2011 to 2021. Front Bioeng Biotechnol. 2022;10:904629. doi: 10.3389/fbioe.2022.904629 35677303 PMC9168279

[15] González-Torres T, Rodríguez-Sánchez JL, Montero-Navarro A, et al. Visualizing research on industrial clusters and global value chains: a bibliometric analysis. Front Psychol. 2020;11:11 1754. doi: 10.3389/fpsyg.2020.01754 32793074 PMC7387649

[16] Shen Y, Qu W, Yu F, et al. Dynamic associations between the respiratory tract and gut antibiotic resistome of patients with COVID-19 and its prediction power for disease severity. Gut Microbes. 2023;15:2223340. doi: 10.1080/19490976.2023.2223340 37306468 PMC10262814

[17] Fais F, Juskeviciene R, Francardo V, et al. Drug-free nasal spray as a barrier against SARS-CoV-2 and its delta variant: in vitro study of safety and efficacy in human nasal airway epithelia. Int J Mol Sci. 2022;23:4062. doi: 10.3390/ijms23074062 35409423 PMC8999825

[18] de lira-Quezada CE, González-Díaz SN, Cotera-de Lira AG, et al. The association of air pollution in respiratory allergy: its impact in an industrial city. World Allergy Organ J. 2024;17:100867. doi: 10.1016/j.waojou.2023.100867 38370131 PMC10869943

[19] Brożek JL, Bousquet J, Agache I, et al. Allergic rhinitis and its impact on asthma (ARIA) guidelines-2016 revision. J Allergy Clin Immunol. 2017;140:950–958. doi: 10.1016/j.jaci.2017.03.050 28602936

[20] Chen H, Meng X, Yu Y, et al. Greenness and its composition and configuration in association with allergic rhinitis in preschool children. Environ Res. 2024;251:118627. doi: 10.1016/j.envres.2024.118627 38460662

[21] Lu C, Zhang Y, Li B, et al. Interaction effect of prenatal and postnatal exposure to ambient air pollution and temperature on childhood asthma. Environ Int. 2022;167:167 107456. doi: 10.1016/j.envint.2022.107456 35952466

[22] Shi H, Wang T, Zhao Z, et al. Prevalence, risk factors, impact and management of pneumonia among preschool children in Chinese seven cities: a cross-sectional study with interrupted time series analysis. BMC Med. 2023;21:227. doi: 10.1186/s12916-023-02951-2 37365601 PMC10294363

[23] Schraufnagel DE, Balmes JR, Cowl CT, et al. Air pollution and noncommunicable diseases: a review by the forum of international respiratory societies’ environmental committee, part 2: air pollution and Organ systems. Chest. 2019;155:417–426. doi: 10.1016/j.chest.2018.10.041 30419237 PMC6904854

[24] Celebi Sozener Z, Ozdel Ozturk B, Cerci P, et al. Epithelial barrier hypothesis: effect of the external exposome on the microbiome and epithelial barriers in allergic disease. Allergy. 2022;77:1418–1449. doi: 10.1111/all.15240 35108405 PMC9306534

[25] van den Bosch M, Meyer-Lindenberg A. Environmental Exposures and depression: biological mechanisms and epidemiological evidence. Annu Rev Public Health. 2019;40:239–259. doi: 10.1146/annurev-publhealth-040218-044106 30633709

[26] Shabani Isenaj Z, Berisha M, Gjorgjev D, et al. Air pollution in Kosovo: short term effects on hospital visits of children due to respiratory health diagnoses. Int J Environ Res Public Health. 2022;19:10141. doi: 10.3390/ijerph191610141 36011773 PMC9407926

[27] Ranasinghe D, Lee ES, Zhu Y, et al. Effectiveness of vegetation and sound wall-vegetation combination barriers on pollution dispersion from freeways under early morning conditions. Sci Total Environ. 2019;658:658 1549–1558. doi: 10.1016/j.scitotenv.2018.12.159 PMC7092696 30678013

[28] Kim BJ, Hong SJ. Ambient air pollution and allergic diseases in children. Korean J Pediatr. 2012;55:185–192. doi: 10.3345/kjp.2012.55.6.185 22745642 PMC3382698

[29] Liu X, Xiao Y, Zhu Q, et al. Circulating endothelial progenitor cells are preserved in female mice exposed to ambient fine particulate matter independent of estrogen. Int J Mol Sci. 2021;22:7200. doi: 10.3390/ijms22137200 34281260 PMC8268796

[30] Eckl-Dorna J, Klein B, Reichenauer TG, et al. Exposure of rye (secale cereale) cultivars to elevated ozone levels increases the allergen content in pollen. J Allergy Clin Immunol. 2010;126:1315–1317. doi: 10.1016/j.jaci.2010.06.012 20674963

[31] D’Amato G, D’Amato M. Climate change, air pollution, pollen allergy and extreme atmospheric events. Curr Opin Pediatr. 2023;35:356–361. doi: 10.1097/MOP.0000000000001237 36917187

[32] Ščevková J, Dušička J, Zahradníková E, et al. Impact of meteorological parameters and air pollutants on airborne concentration of betula pollen and bet v 1 allergen. Environ Sci Pollut Res Int. 2023;30:95438–95448. doi: 10.1007/s11356-023-29061-z 37544949 PMC10482788

[33] Sedghy F, Varasteh AR, Sankian M, et al. Interaction between air pollutants and pollen grains: the role on the rising trend in allergy. Rep Biochem Mol Biol. 2018;6:219–224. 29766006 PMC5941124

[34] Eguiluz-Gracia I, Mathioudakis AG, Bartel S, et al. The need for clean air: the way air pollution and climate change affect allergic rhinitis and asthma. Allergy. 2020;75:2170–2184. doi: 10.1111/all.14177 31916265

[35] Annesi-Maesano I. The air of Europe: where are we going? Eur Respir Rev. 2017;26:170024. doi: 10.1183/16000617.0024-2017 29212835 PMC9488903

[36] Watts N, Amann M, Arnell N, et al. The 2018 report of the lancet countdown on health and climate change: shaping the health of nations for centuries to come. Lancet. 2018;392:2479–2514. doi: 10.1016/S0140-6736(18)32594-7 30503045 PMC7616804

[37] D’Amato G, Annesi-Maesano I, Cecchi L, et al. Latest news on relationship between thunderstorms and respiratory allergy, severe asthma, and deaths for asthma. Allergy. 2019;74:9–11. doi: 10.1111/all.13616 30242857

[38] Cramer J, Jørgensen JT, Hoffmann B, et al. Long-term exposure to air pollution and incidence of myocardial infarction: a Danish nurse cohort study. Environ Health Perspect. 2020;128:57003. doi: 10.1289/EHP5818 32438827 PMC7263451

